# Drug delivery system of curcumin to the lungs based on poly(3-alloxyloxy-1,2-propylene succinate)-sebacic acid copolymers

**DOI:** 10.1016/j.jpha.2025.101434

**Published:** 2025-08-14

**Authors:** Karolina Knap, Konrad Kwiecień, Jonasz Czajkowski, Rafał Szostecki, Daria Niewolik, Katarzyna Jaszcz, Peter Olinga, Katarzyna Reczyńska-Kolman, Elżbieta Pamuła

**Affiliations:** a Department of Biomaterials and Composites, Faculty of Materials Science and Ceramics, AGH University of Krakow, Kraków, 30-059, Poland; b Department of Physical Chemistry and Technology of Polymers, Faculty of Chemistry, Silesian University of Technology, Gliwice, 44-100, Poland; c Groningen Research Institute of Pharmacy, Pharmaceutical Technology and Biopharmacy, University of Groningen, Groningen, 9713 AV, the Netherlands

**Keywords:** Polyanhydrides, Microparticles, Curcumin, Drug delivery systems, Pulmonary delivery

## Abstract

Polyanhydrides are attractive materials for drug delivery matrices as a result of their cytocompatibility and fast degradation rate. Here, we synthesized and characterized copolymers of poly(3-allyloxy-1,2-propylene succinate) (PSAGE) and sebacic acid (SBA). The successful polymerization was confirmed by proton nuclear magnetic resonance (^1^H NMR) and Fourier transform infrared (FTIR) spectroscopy analyses. The material with PSAGE and 60% of SBA copolymer (PSAGE-SBA60) was more hydrophilic than the PSAGE and 80% of SBA copolymer (PSAGE-SBA80) (water contact angle 82.2° ± 11.6° vs. 98.6° ± 8.9°, respectively). PSAGE-SBA60 also had a lower molecular weight than PSAGE-SBA80 (M_n_ = 6400 Da vs. 9800 Da). Both polyanhydrides were used to encapsulate curcumin (CUR) as a potential anti-inflammatory, antimicrobial and anticancer agent. The unloaded microparticles (MPs) and CUR-loaded MPs were produced using the emulsification/solvent evaporation method. The CUR was uniformly distributed within the MPs, as confirmed by fluorescence microscopy. All MPs had a geometric diameter < 5 μm and their surface charge was negative. MPs_PSAGE-SBA80 + CUR had the best aerodynamic properties, as shown by laser diffraction measurements and flowability parameters, i.e., Carr index and Hausner ratio. The MPs obtained from PSAGE-SBA60 degraded faster than those of PSAGE-SBA80. All MPs were noncytotoxic at a concentration of up to 100 μg/mL in the *in vitro* model (BEAS-2B lung epithelial cells) and *ex vivo* precision-cut tissue slices (PCTSs) rat model. The developed MPs are promising CUR carriers for pulmonary delivery in a dry powder formulation.

## Highlights

• Curcumin-loaded poly(ester-anhydride) microparticle formulation for inhalation.
• Formulation with appropriate size, degradation rate, and aerodynamic properties to be deposited in the lungs.
• Microparticles cytocompatible up to 100 μg/mL according to *in vitro* and *ex vivo* models.

## Graphical abstract

**Figure:**
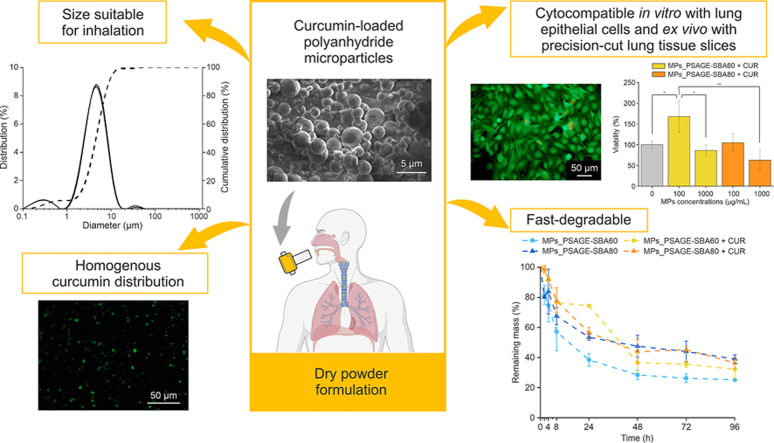

## 1 Introduction

Lung diseases are one of the leading causes of morbidity and mortality worldwide, leading to an increased economic and social burden. The most common diseases include chronic obstructive pulmonary disease (COPD), asthma, acute lower respiratory tract infections, tuberculosis, and lung cancer [1]. Their treatment can be improved by delivering active pharmaceutical ingredients (APIs) encapsulated in microparticles (MPs) directly to the lungs [2]. Providing APIs by inhalation reduces the total doses of APIs administered to the patient, decreases the frequency of the dosage and the onset of action, and reduces the risk of API inactivation, e.g., during first-pass metabolism in the liver [3,4]. However, this route of delivery is not free from limitations. In the respiratory tract, MPs are easily removed by mucociliary clearance [5] or by macrophage phagocytosis [6]. Thus, fast degradation of the carrier and rapid drug release are required. Furthermore, it is necessary to obtain MPs with a particle size in the range of 1–5 μm, which allows them to be deposited in the lower respiratory tract [4]. Inhalable carriers can be used for encapsulation of various synthetic drugs, e.g., anticancer [[5], [6], [7]], antibacterial [[8], [9], [10]], or anti-inflammatory [11,12].

Alternatively to synthetic drugs, a variety of natural anti-inflammatory and antibacterial moieties can be encapsulated within inhalable carriers. Polyphenols, compounds with at least one aromatic ring and at least two hydroxyl groups, occur naturally in plants and are well known for their biological activity [13]. In addition, these substances are attractive as potential agents for the prevention and treatment of diseases related to oxidative stress. Furthermore, polyphenols have other biological properties, such as cardioprotective, anticancer, anti-ageing, and antimicrobial [14]. Mahar et al. [15] encapsulated resveratrol (RSV) in polycaprolactone MPs to treat COPD. The study demonstrated that encapsulated RSV has better aerodynamic properties compared to pure RSV administered by dry powder inhalation. Additionally, they confirmed that MPs have antioxidant properties.

Herein, we have chosen curcumin (CUR), one of the polyphenols most often considered in the field of drug delivery, for encapsulation in polyanhydride MPs. Hu et al. [16] encapsulated CUR in poly(lactic-*co*-glycolic acid) (PLGA) to treat idiopathic pulmonary fibrosis. The obtained MPs have been shown to release CUR before uptake by macrophages and have anti-inflammatory properties. PLGA is a widely used copolymer; however, it has some limitations as a pulmonary drug carrier. Depending on the molecular weight and the molar ratio of lactic acid to glycolic acid, PLGA degrades within three weeks to more than a year. Natural clearance mechanisms have limited capabilities, which is why the slow degradation of PLGA could result in accumulation of MPs in the lungs. As a result, other copolymers have been tested as pulmonary drug carriers. Lai et al. [17] synthesized two copolymers: one of them was obtained from methoxy poly(ethylene glycol) (mPEG) and polylactide (PLLA), while the second one was synthesized from mPEG and polyanhydride, i.e., poly(sebacic acid) (PSBA). CUR-loaded micelles were prepared from both copolymers. The results showed that the micelles obtained from mPEG-PSBA had better encapsulation efficiency (EE) and drug loading (DL), and released CUR significantly faster compared to the mPEG-PLLA micelles. The results suggest that polyanhydrides may be suitable for pulmonary drug delivery. Polyanhydrides have unique properties, such as controlled biodegradability, zero-order release kinetics, fast, hydrolytic and surface degradation, and low toxicity of degradation products [18]. Despite the potential of polyanhydrides, there are only a few publications focussing on the manufacturing and characterization of polyanhydride-based MPs as pulmonary carriers. Fiegel et al. [19] obtained empty MPs from copolymers of poly(ethylene glycol) (PEG) and PSBA. The MPs had an optimal size for pulmonary drug delivery; however, the influence of APIs on the properties and cytotoxicity of the MPs was not evaluated. In the other study, Niewolik et al. [20] manufactured MPs from betulin and PEG-based polyanhydrides and confirmed that MPs can be obtained from polyanhydrides with good aerodynamic properties. According to the literature, all the studies reported so far have been focused on empty MPs, without any encapsulated API.

The aim of this study was to synthesize and characterize copolymers of poly(3-allyloxy-1,2-propylene succinate) (PSAGE) and sebacic acid (SBA). The success of polycondensation was confirmed by proton magnetic nuclear resonance (^1^H NMR) and Fourier transform infrared (FTIR) spectroscopy. The molecular mass, thermal properties, hydrophobicity, and surface free energy (SFE) of the materials were also determined. From these materials, the MPs loaded with CUR were prepared by oil-in-water emulsification, and their size, physicochemical and aerodynamic properties, as well as cytotoxicity, were evaluated. To the best of our knowledge, this is the first report on CUR encapsulation in PSAGE-SBA copolymers and their characterization as potential carriers for pulmonary delivery.

## 2 Materials and methods

### 2.1 Synthesis and characteristics of copolymers

#### 2.1.1 Synthesis of PSAGE and SBA copolymers

PSAGE was synthesized by melt condensation of succinic acid (SAc) (Sigma-Aldrich, St. Louis, MO, USA) and allyl glycidyl ether (AGE) (Sigma-Aldrich) using twofold excess of SAc according to the previously described protocol [21]. Briefly, the reaction was carried out at 150 °C until the acid value (AV) decreased to a constant value. The crude product was dissolved in chloroform (STANLAB, Lublin, Poland) and filtered to remove unreacted SAc. The oligoester was precipitated in a mixture of diethyl ether (Chempur GmbH, Piekary Śląskie, Poland) and petroleum ether (1:1, *V*/*V*) (Chempur GmbH), separated by sedimentation, and dried under vacuum (0.4 kPa). 3-Allyloxy-1,2-propylene succinate (OSAGE) or SBA (Sigma-Aldrich) were refluxed in acetic anhydride (1:10, *m*/*V*) under nitrogen for 30 min. Excess of acetic anhydride and acetic acid formed as a by-product were removed under vacuum. The prepolymers obtained were dissolved in methylene chloride (Chempur GmbH), precipitated in the mixture of diethyl ether and petroleum ether (1:1, *V*/*V*), separated, and dried under a vacuum. The prepolymers were stored at −18 °C. PSAGE and SBA prepolymers were then mixed in defined ratios (40:60 (*m*/*m*) and 20:80 (*m*/*m*) for PSAGE-SBA60 and PSAGE-SBA80, respectively), stirred with a magnetic stirrer (M57-H550-PRO, Onilab LLC, San Francisco, CA, USA) and heated at 150 °C for 2 h under high vacuum conditions (0.0133–0.0013 kPa) to yield poly(ester-anhydride)s. The poly(ester-anhydride)s obtained were dissolved in methylene chloride, precipitated in diethyl ether/petroleum ether (1:1, *V*/*V*), washed with petroleum ether, and dried under vacuum. The polymers obtained were stored at −20 °C.

#### 2.1.2 Proton nuclear magnetic resonance (^1^H NMR)

^1^H NMR spectroscopy was used to evaluate the polycondensation efficiency. The ^1^H NMR spectra of polymers in CDCl_3_ (Deutero GmbH, Kastellaun, Germany) were recorded on a 300 MHz spectrometer with tetramethylsilane (TMS) as an internal standard (Varian Medical Systems, Palo Alto, CA, USA).

#### 2.1.3 FTIR spectroscopy

The chemical composition of the synthesized copolymers was evaluated by FTIR (Tensor 27; Bruker, Billerica, MA, USA). Briefly, copolymers were dried overnight in a vacuum drier (Vacucell 5S Comfort; MMM Group, München, Germany) prior to the experiment. Then each sample was homogenized with 200 mg of KBr (Sigma-Aldrich) using an agate mortar. The resulting powders were compressed into pellets at 740 MPa pressure using a pellet die with a manual hydraulic press (Specac, Orpington, UK), and the IR absorbance was measured in transmittance mode for the wavenumbers of 4000–400 cm^−1^ (32 scans, resolution 4 cm^−1^). A pure KBr pellet was used to register the background. An automatic baseline correction was performed (number of baseline points, 64 and number of iterations, 10). The data were analysed using the OPUS 7.2 software (2012).

#### 2.1.4 Gel permeation chromatography (GPC)

GPC was used to determine the molecular weight and molecular weight distribution. Measurements were made in methylene chloride at a flow rate of 0.8 mL/min using a chromatograph with a refractive index detector (Infinity 1260; Agilent Technologies, Santa Clara, CA, USA). The chromatograph was calibrated using linear polystyrene standards (580–300,000 Da).

#### 2.1.5 Differential scanning calorimetry (DSC)

The analysis of thermal stability and crystallinity was determined by DSC (DSC1; Mettler Toledo, Columbus, OH, USA). Polymers (approximately 4 mg) were placed in pierced aluminium crucibles. The test was carried out in nitrogen at a flow rate of 30 mL/min in heating/cooling/heating cycles in a temperature range of −90 to 160 °C at a heating/cooling rate of 10 °C/min.

#### 2.1.6 Wettability and SFE

Wettability and SFE were determined using the sessile drop technique with MilliQ water (Direct-Q3UV, Merck Millipore; Burlington, MA, USA) (polar liquid) and CH_2_I_2_ (Avantor Performance Materials Poland S.A., Gliwice, Poland) (nonpolar liquid). To obtain a flat surface for the measurements, polymers were deposited on 12 mm diameter glass coverslips. To do so, the copolymers were dissolved in dichloromethane (DCM) (POCH) at a concentration of 50 mg/mL. The glass coverslips were then placed in a container of the same diameter and 2 mL of each solution was poured into the containers. The samples were left in the fume hood until the solvent was completely evaporated. The homogeneous polymer layers obtained were used for wettability analysis (DSA10 goniometer; Krüss Scientific, Hamburg, Germany). For each liquid, 3 droplets were deposited on 2 separate samples of each material. SFE calculations were performed based on the Owens, Wendt, Rabel, and Kaelble (OWRK) model using drop shape analysis software (Krüss Scientific).

### 2.2 Manufacturing and characteristics of MPs

#### 2.2.1 MPs manufacturing

MPs were obtained using a modification of the previously described method [22,23]. Briefly, 2% (*m*/*V*) solutions of the copolymers in DCM were prepared. Beakers with 30 mL of 8% (*m*/*V*) poly(vinyl alcohol) (PVA) (Moviol 8–44; Sigma-Aldrich) dissolved in MilliQ water were placed in crystallizers with ice. The oil phase was instilled into the water phase at a stirring speed of 1500 rpm (MS-52 M; Jeio Tech Co., Ltd., Daejeon, Republic of Korea). The stirring continued for 2 h to evaporate the organic solvent. The suspensions were then centrifuged (15,000 rpm, 6 min) (MPW-351R; MPW MED. INSTRUMENTS, Warsaw, Poland). The supernatants were discarded and the MPs were resuspended in the Milli-Q water and centrifuged three times. After discarding the supernatant, the MPs were frozen at −80 °C for 24 h and then freeze dried for 48 h (Alpha 1–2 LD plus; Martin Christ Gefriertrocknungsanlagen GmbH, Osterode am Harz, Germany). The CUR loaded MPs were prepared using an analogous protocol with some modifications: 8 mg of CUR (Sigma-Aldrich) were suspended in 2 mL of each polymer solution by ultrasound homogenization (time, 90 s; pulse, 20 s on and 5 s off; and amplitude, 40%) (Vibra-Cell^TM^; Sonics & Materials Inc., Newtown, CT, USA) and then instilled in the water phase. The dried MPs were stored at −20 °C prior to further tests.

#### 2.2.2 Scanning electron microscopy (SEM)

The shape and morphology of the MPs were assessed by SEM (Apreo 2; Thermo Fisher Scientific Inc., Waltham, MA, USA). The MPs were placed on standard aluminium SEM holders, attached with carbon tape, and coated with a 5-nm thick gold layer (EM ACE 600; Leica, Wetzlar, Germany). The images were taken at a magnification of 20,000 times. The size distribution of the MPs was determined using ImageJ software, and the diameter of 200 individual MPs was measured for each type of MP.

#### 2.2.3 Degradation during the manufacturing process

Because polyanhydrides are prone to hydrolysis even in short contact with water, ^1^H NMR analysis was used to assess the degradation of anhydride groups during the production of empty MPs. Polymers and empty MPs were dissolved in CDCl_3_ at 1 mg/mL. The ^1^H NMR spectra were recorded on a 300 MHz spectrometer with TMS as an internal standard. Data were analysed with MestReNova v6.0.2–5475 software. To evaluate the degree of degradation, we calculated integrals of peaks coming from protons neighbouring the bonded and unbonded −COOH groups of SBA. The ratio of the signal from the anhydride peak to the total signal of the proton type was used to calculate the proportion of bound carboxyl groups. The ratio of the percentage of bonded carboxylic groups before and after the process was used to determine the degradation that occurred during the manufacturing of the MPs.

#### 2.2.4 Optical and fluorescent microscopy

The MPs were observed under optical and fluorescence microscopes. Because of the fluorescent properties of the CUR, it was possible to observe the presence of the CUR inside the MPs. First, the MPs were observed under optical, and then in the same place under fluorescence microscopy. To excite the CUR in the samples, light at 450–480 nm was used. The photographs were taken with an Axiovert 40 CFL inverted microscope (Zeiss, Jena, Germany) equipped with a Kübler Codix HXP 120C fluorescent light source (Zeiss).

#### 2.2.5 Zeta potential

The suspension of the MPs in MilliQ water at 1 mg/mL was placed in dedicated cuvettes (DTS1070) and the surface zeta potential was measured three times (15 runs for each measurement) (ZetaSizer Nano ZS; Malvern Instruments, Malvern, UK).

#### 2.2.6 EE and DL

The EE and DL capacities were determined using the fluorometric method. MPs were dissolved in dimethyl sulfoxide (DMSO) (POCH) at 1 mg/mL solutions and diluted 10-fold to reach the concentration within the linear range of the calibration curve. Three aliquots of each sample were added to the wells of a black 96-well plate and measured using FLUOstar® Omega (BMG Labtech, Ortenberg, Germany) at excitation and emission wavelengths in the range 485–412 nm and 590–510 nm, respectively. The calibration curve was fitted and unknown CUR concentrations were determined. EE and DL were calculated using Eqs. (1), (2)), respectively:

(1) $$\begin{array}{c} \text{EE}\%\;=\;\frac{\text{Encapsulated}\;\text{drug}\;\text{mass}}{\text{Total}\;\text{drug}\;\text{mass}}\;\times \;100\%\; \end{array}$$

(2) $$\begin{array}{c} \text{DL}\%\;=\;\frac{\text{Encapsulated}\;\text{drug}\;\text{mass}}{\text{Microparticles}\;\text{mass}}\;\times \;100\%\; \end{array}$$

#### 2.2.7 FTIR spectroscopy

The CUR loading in MPs was also confirmed by attenuated total reflectance FTIR (ATR-FTIR) spectroscopy using Tensor 27 (Bruker). Dry MPs were placed on a diamond ATR crystal. The spectra were registered in the wavenumber range of 4000–400 cm^−1^ by averaging 64 scans with a resolution of 4 cm^−1^.

#### 2.2.8 Aerodynamic properties of MPs

##### 2.2.8.1 Bulk and tapped density

The bulk density was estimated using a previously published protocol [20]. MPs were loaded into a 1.5 mL polypropylene flask. The mass of MPs and the volume were measured. Then the flask was tapped 100 times on the desk. The procedure was repeated three times for each sample. Bulk density was calculated as the mass divided by the volume of powder before tapping, while the tapped density was calculated as the mass divided by the volume of powder after tapping.

##### 2.2.8.2 Theoretical aerodynamic diameter

The theoretical aerodynamic diameter was calculated using the equation (Eq. 3):

(3) $$d_{aero}\;=\;{\;d}_{g}\sqrt{\frac{\rho_{tapped}}{\chi \cdot \rho_{0}}}$$

where *d*_*aero*_ is theoretical aerodynamic diameter, *d*_*g*_ is the geometric diameter measured based on SEM images, *χ* is shape factor (for spherical MPs is equal to 1), *ρ*_*tapped*_ is tapped density of MPs, and *ρ*_*0*_ is the reference density equal to 1 g/cm^3^.

##### 2.2.8.3 Carr index and Hausner ratio

The Carr index and Hausner ratio were calculated based on bulk and tapped density using the equations (Eqs. (4), (5)), respectively):

(4) $$\text{Carr}\;\text{index }=\;\frac{\rho_{tapped}\;-\;\rho_{bulk}}{\rho_{tapped}}\;\times \;100\%$$

(5) $$\text{Hausner}\;\text{ratio }=\;\frac{\rho_{tapped}}{\rho_{bulk}}$$

where *ρ*_*tapped*_ is tapped density of MPs and *ρ*_*bulk*_ is bulk density of MPs. The flow character was determined according to (Table 1) [24].

**Table 1:** The flow character of microparticles (MPs) [24].

Flow character of powders	Hausner ratio	Carr index
Excellent	1.00–1.11	≤10
Good	1.12–1.18	11–15
Fair	1.19–1.25	16–20
Passable	1.26–1.34	21–25
Poor	>1.35	>26

##### 2.2.8.4 Laser diffraction measurements

The diameter of the dried particles was measured using a laser diffraction method (MasterSizer 3000E; Malvern Instruments). The feed pressure was set to 4 bars and the feed rate was precisely adjusted to maintain optimal levels of obscuration. For each sample, two independent measurements were made. The one measured lasted 3 s, with the machine consistently administering the particle powder throughout the process. The software was configured to collect data only when the laser obscuration was in the proper range (0.5%–6%), as advised by the producer.

#### 2.2.9 Degradation study

The degradation rate was determined on the basis of the mass loss of the MPs and the change in pH of the incubation medium. The MPs were incubated in phosphate-buffered saline (PBS) (Sigma-Aldrich) at 37 °C for 96 h. MPs suspensions in PBS (1 mg/mL) in Eppendorf tubes (Eppendorf, Hamburg, Germany) were placed in an incubator and gently shaken (50 rpm; ROTATOR MUL-TIMIX; VWR Corporation, Radnor, PA, USA). At predetermined time points, the MPs were centrifuged (18,000 rpm, 4 min) and the supernatants were separated from the MPs. The latter were rinsed with MilliQ water three times and centrifuged. Then the MPs were frozen, freeze-dried, and weighed. The pH values of the supernatants were measured using a pH-meter (CP-401; Elmetron, Zabrze, Poland). All measurements were taken after 2, 4, 8, 24, 48, 72, and 96 h.

### 2.3 Cytotoxicity *in vitro*

Cytotoxicity of the MPs was evaluated *in vitro* in contact with human lung epithelial cells (BEAS-2B, CRL-3588; American Type Culture Collection (ATCC), Manassas, VA, USA). Cells were cultured in Dulbecco's modified Eagle's medium (DMEM) (PAN-Biotech, Aidenbach, Germany) supplemented with 10% fetal bovine serum (FBS) (PAN-Biotech) and 1% penicillin/streptomycin (PAN-Biotech). Cell culture was carried out in a humidified atmosphere at 37 °C, with 5% CO_2_. The cells were seeded in the 96-well plate, at 10,000 cells/well, and incubated for 24 h in 100 μL of medium. The MPs were sterilized under ultraviolet (UV) light for 20 min and dispersed in the medium at different concentrations, i.e., 0 (control sample), 5, 10, 50, 100, 500, and 1000 μg/mL. The MPs were added to the previously seeded cells (100 μL of MPs suspension in triplicate) for 24 h. The viability of the cells was determined using the resazurin reduction assay and live/dead fluorescence staining. The cytotoxicity of the CUR-loaded MPs was investigated in an analogical way.

A resazurin reduction assay was performed to assess the metabolic activity of the cells. The medium containing the MPs was removed and the cells were rinsed three times in 100 μL PBS (PAN-Biotech) to remove the residual MPs. Then 150 μL of 10% AlamarBlue solution (resazurin sodium salt; Sigma-Aldrich) dissolved in PBS at a concentration of 0.1 mg/mL and then added into the medium at 10% (*V*/*V*) concentration, was added to the cells and incubated for 3 h. Then 100 μL of each well content was transferred to a black 96-well plate and the fluorescence signal was measured using the microplate reader at excitation and emission wavelengths equal to 544 and 590 nm, respectively. The viability of cells was calculated as the fluorescence of the sample divided by the mean fluorescence of the control sample.

The medium containing the MPs was discarded and the cells were rinsed with PBS to remove residual MPs. 100 μL of a mixture containing 0.1% calcein acetoxymethyl ester (AM) (Sigma-Aldrich) and 0.1% propidium iodide (Sigma-Aldrich) in PBS was added to each well. After 20 min of incubation at 37 °C in the dark, images were taken using fluorescence microscopy as described in Section 2.2.4.

### 2.4 Cytotoxicity *ex vivo*

*Ex vivo* experiments were carried out using precision-cut tissue slices (PCTSs) from the lungs of male Wistar rats (Crl:WI; Charles River Laboratories‌, Wilmington, MA, USA). Animals were sacrificed by exsanguination through the abdominal aorta. Three rats were used independently for repetitions of the same experiment. The termination was performed on different days but at similar hours to avoid variation caused by the circadian rhythm. After sacrifice of the animals, the lungs were intratracheally inflated with a solution of 1.5% agarose (Sigma-Aldrich, Zwijndrecht, The Netherlands) and 0.9% NaCl (Merck; Darmstadt, Germany). Subsequently, the lungs were harvested and placed in the University of Wisconsin solution (UW) (DuPont, Waukegan, IL, USA) with constant cooling in ice. To obtain the PCTS, the lung lobes were separated, and cylindrical tissue samples were excised using a 5 mm biopsy puncher. The samples prepared were washed with fresh UW; and then cut into thin slices (weighting 4–5 mg) in the Krebs solution (2.1 g NaHCO_3_ (Merck), 4.5 g glucose (Merck), and 2.38 g 4-(2-hydroxyethyl)-1-piperazineethanesulfonic acid (HEPES) (VWR Corporation) per 1 L of MiliQ-water) using Krumdieck Tissue Slicer (Alabama Research and Development, Munford, TN, USA). The PCTS were placed again in the fresh UW.

MPs suspensions were obtained in an analogous way to those dedicated to *in vitro* cytotoxicity tests. However, a different culture medium was used (Advanced DMEM/F-12 (Thermo Fisher Scientific Inc.) supplemented with 200 mM GlutaMAX (Thermo Fisher Scientific Inc.), 1M HEPES, 10,000 U/mL penicillin-streptomycin mix (Thermo Fisher Scientific Inc.), and 50 mg/mL gentamycin (Thermo Fisher Scientific Inc.). Solutions of concentrations of 0, 1, 10, 100, and 1000 μg/mL of both PSAGE-SBA60 and PSAGE-SBA80 MPs were pre-incubated for 1 h at 37 °C and 5% CO_2_ in 12-well cell culture plates (1.3 mL each, in triplicates for each rat) to adjust temperature and pH. The PCTS (three per each sample type) were then placed in the suspensions and incubated for 24 h under the same conditions with constant shaking. Subsequently, the samples were collected and placed in 1 mL of the sonification solution containing 70% ethanol and 2 mM ethylenediaminetetraacetic acid (EDTA) (Merck) (70:30, *V*/*V*) (pH 10.9) with glass beads and frozen in liquid nitrogen. Until further examination, the samples were stored at −80 °C.

The influence of the presence of the MPs on the viability of the PCTS was analysed using the adenosine-triphosphate (ATP) assay according to previously established protocols [25,26]. Briefly, the samples were gradually defrosted and kept at 4 °C to avoid degradation of ATP. Intensive shaking was introduced (2 cycles for 45 s) to decompose the PCTS (Mini-BeadBeater; BioSpec, Bartlesville, OK, USA). The samples were then centrifuged at 13,000 rpm for 5 min (Eppendorf). 5 μL of each supernatant was diluted in 45 μL of tris-EDTA buffer (100 mM tris(hydroxymethyl)amniophen and 2 mM EDTA (pH 7.6−8.0, adjusted with HCl (both from Merck)) and placed in a white 96-well plate. Afterwards, 50 μL of luciferase (ATP Bioluminescence Assay Kit; Roche, Basel, Switzerland) was added to each well. Luminescence (BioTek Synergy HT; Marshall Scientific, Hampton, NH, USA) was measured immediately after the reagent was added. The ATP levels were calculated on the basis of the ATP standard solution (ATP Bioluminescence Assay Kit; Roche), whose luminescence was measured analogically. All samples (three for each sample type for each rat) were measured in duplicates (18 measurements per sample total).

To compare the PCTS, their ATP levels were normalized using the total amount of protein within the samples. To do so, the samples after the ATP assay were left to dry at 37 °C overnight, and then, the dry residues were dissolved again in 200 μL of 5M NaOH (Merck) by 30 s of intensive shaking each. The samples were then diluted with 800 μL of MilliQ water and shaken again for 45 s. 5 μL of each sample was placed in a transparent 96-well plate. Afterwards, reagents A and B from RC DC Protein Assay Kit (Bio-Rad, Munich, Germany) (25 and 200 μL, respectively) were added and the absorbance at 655 nm was measured after 15 min of incubation. For protein level calculations, known concentrations of bovine serum albumin (BSA) (Sigma-Aldrich), as standard protein were used for calibration. All measurements were carried out in duplicate.

All animal experiments were approved by the he Animal Ethical Committee of the University of Groningen (Groningen, The Netherlands) (Common Core Definition (CCD) Approval No.: AVD10500202216104) and were performed in accordance with EU Directive 2010/63/EU for animal experiments.

### 2.5 Statistical analysis

The statistical analyses of the obtained data were done using a one-way analysis of variance (ANOVA) followed by Tuckey's post hoc test. The analyses were performed using OriginLab software. The results are presented as mean ± standard deviation (SD).

## 3 Results

### 3.1 Copolymers characterization

**Fig. 1:**
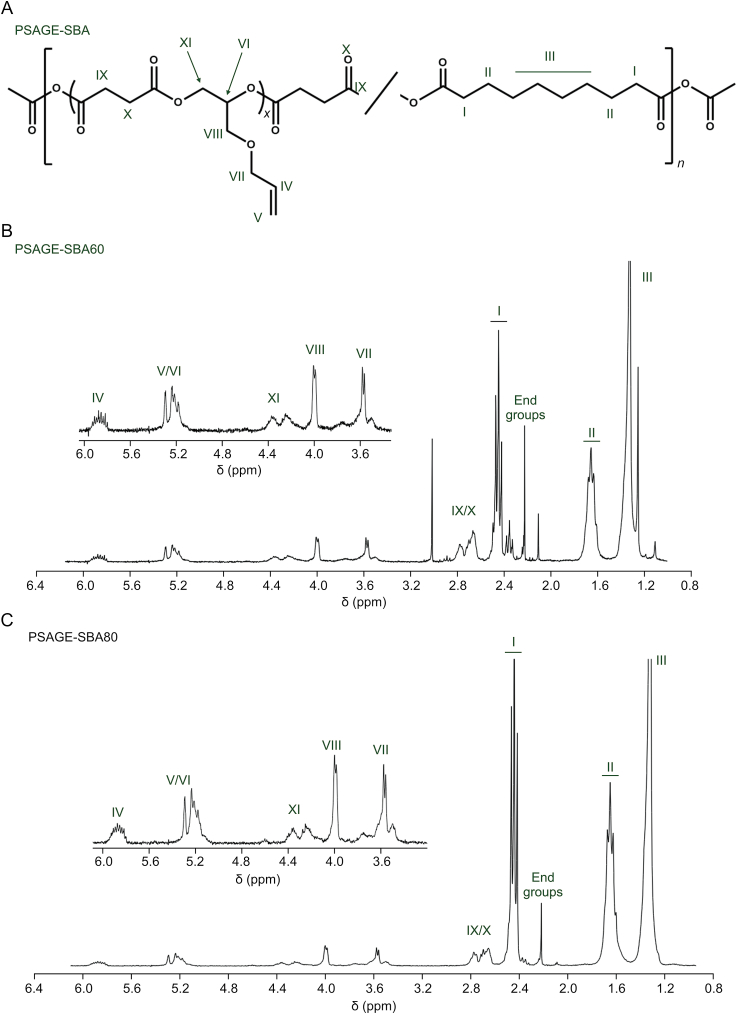
Chemical structure and chemical analysis of synthesized polyanhydrides. (A) Structure of poly(3-allyloxy-1,2-propylene succinate) (PSAGE) and sebacic acid (SBA) copolymer (PSAGE-SBA). (B) Proton magnetic nuclear resonance (^1^H NMR) spectra of PSAGE and 60% of SBA copolymer (PSAGE-SBA60). (C) ^1^H NMR spectra of PSAGE and 80% of SBA copolymer (PSAGE-SBA80). The insets indicate the spectra at higher magnification in the range of 3.3–6.1 ppm. Signal assignment: I and II, methyl groups; III, aliphatic chain of SBA; IV–VI, hydrogen atoms attached to the unsaturated atom of the end of the side chain; VII and VIII, peaks from ether-neighbouring protons in 3-allyoxy-1,2-propylene; IX and X, methyl groups in succinic acid (SAc); and XI, hydrogen atoms in 3-allyoxy-1,2-propylene bonded to the oxygen of the ester group and the branching hydrocarbon.

The copolymers of PSAGE and SBA were successfully synthesized by polycondensation. For synthesis, PSAGE and SBA were mixed in different feed ratios: 40:60 (*m*/*m*) (PSAGE-SBA60) and 20:80 (*m*/*m*) (PSAGE-SBA80) (Fig. 1A).

The structure of the PSAGE-SBA copolymers was confirmed by ^1^H NMR (Figs. 1B and C). The signal from the aliphatic chain of SBA was identified in the range of 1.50 to 1.20 ppm (III). The signal of the methyl groups was shifted to higher values of 1.80–1.50 ppm (II) and 2.60–2.40 ppm (I) [23]. The methyl groups in SAc are responsible for the double peak appearing between 2.85 and 2.60 ppm (IX/X). The neighbouring groups of the ester or anhydride group are responsible for the small shift. Hydrogen atoms attached to the unsaturated carbon at the end of the side chain produced the highest ppm values, which were 6.0–5.8 ppm (IV) and 5.4–5.1 ppm (V/VI). We can also expect a signal from a proton present in branched hydrocarbons that coincides with the other signal in the region of 5.4 to 5.1 ppm (V/VI). Furthermore, in 3-allyoxy-1,2-propylene, there are peaks from ether-neighbouring protons at 4.0 (VIII) and 3.6 ppm (VII), respectively. The last detected signal is between 4.4 and 4.2 ppm (XI), which is the region where hydrogen atoms in 3-allyoxy-1,2-propylene are bonded to the oxygen of the ester group and the branching hydrocarbon [27,28].

**Fig. 2:**
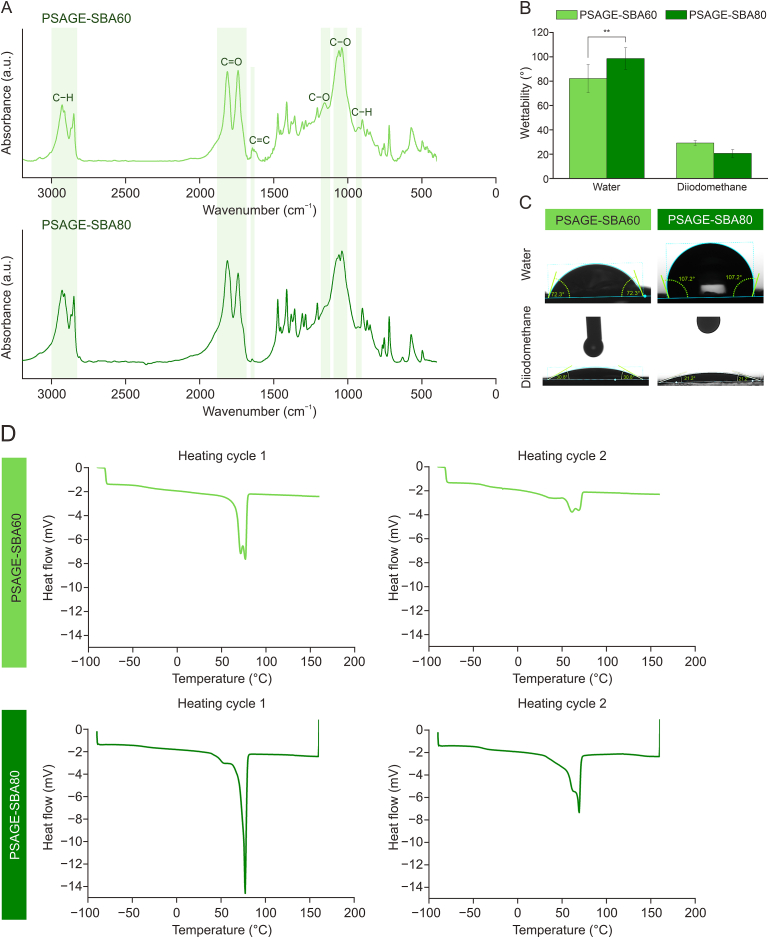
Physicochemical analyses of poly(3-allyloxy-1,2-propylene succinate) (PSAGE) and 60% of sebacic acid (SBA) copolymer (PSAGE-SBA60) and PSAGE and 80% of SBA copolymer (PSAGE-SBA80). (A) Fourier transform infrared (FTIR) spectra. (B) Wettability with water and diiodomethane. (C) Images of the drops. (D) Differential scanning calorimetry (DSC) thermographs of two heating cycles. ^∗∗^*P* < 0.01.

Furthermore, the success of the polycondensation was confirmed by FTIR spectroscopy (Fig. 2A). The bands at 2850–2960 cm^−1^ are from stretching vibrations of C−H bonds in the aliphatic chains of the polymers. The next characteristic bands were observed at 1700–2000 cm^−1^ with two maxima at 1740 and 1810 cm^−1^ due to stretching vibrations of carbonyl groups. For these copolymers, the band at 1740 cm^−1^ is associated with the ester bond, whereas the strong band at 1810 cm^−1^ originates from the stretching vibrations of the carbonyl group in the anhydride bond. The band at 1640–1680 cm^−1^ originates from C=C stretching vibrations. The bands in two regions, 1120–1140 cm^−1^ and 1050–1150 cm^−1^, are due to C−O stretching vibrations from PSAGE used for the synthesis of copolymers or from bonds occurring in anhydrides, esters, acids, and ethers. The last characteristic band at 920–940 cm^−1^ originates from C−H deformation vibrations of aliphatic bonds but only for branched hydrocarbons, which are present in PSAGE in the 3-allyoxy-1,2-propylene part [29,30].

Wettability and SFE were tested using the sessile drop method (Figs. 2B and C). The water contact angle was equal to 82.2^o^ ± 11.6^o^ and 98.6^o^ ± 8.9^o^ for PSAGE-SBA60 and PSAGE-SBA80, respectively. A statistical difference at *P* *<* 0.01 was observed between both materials. However, the difference was not significant between the materials tested with diiodomethane, where contact angles were equal to 29.3^o^ ± 2.1^o^ and 20.1^o^ ± 3.3^o^ for PSAGE-SBA60 and PSAGE-SBA80, respectively. The SFE was similar for both materials and was approximately 47 mJ/m^2^ (Table 2). For PSAGE-SBA60, the polar part of the SFE accounted for 2.8 ± 0.8 mJ/m^2^, while for PSAGE-SBA80, all the energy came from the disperse component and the polar interactions were negligible, although a small increase in the polar interactions was observed for a higher PSAGE ratio. The test showed that the polymers were hydrophobic as expected, and that the one with a higher OSAGE ratio was more hydrophilic. PSAGE-SBA60 showed a higher polar component than PSAGE-SBA80, and this parameter is often associated with more hydrophilic surfaces.

**Table 2:** Surface free energy (SFE), disperse and polar components of SFE of poly(3-allyloxy-1,2-propylene succinate) (PSAGE) and 60% of sebacic acid (SBA) copolymer (PSAGE-SBA60) and PSAGE and 80% of SBA copolymer (PSAGE-SBA80).

Sample	SFE (mJ/m^2^)	Disperse (mJ/m^2^)	Polar (mJ/m^2^)
PSAGE-SBA60	47.3 ± 1.7	44.5 ± 0.9	2.8 ± 0.8
PSAGE-SBA80	47.6 ± 0.8	47.5 ± 0.6	0.1 ± 0.2

The number-average molecular mass (*M*_*n*_), the weight-average molecular mass (*M*_*w*_), and the polydispersity (DP) of PSAGE-SBA60 and PSAGE-SBA80 were determined by the GPC technique (Table 3). PSAGE-SBA60 had lower *M*_*n*_ and *M*_*w*_ than PSAGE-SBA80. Decreased molecular weight of PSAGE-SBA60 was advantageous, to improve its solubility in DCM for an easier MPs manufacturing process. The DP, on the other hand, was similar for both polymers.

**Table 3:** Molecular weight and thermal properties of poly(3-allyloxy-1,2-propylene succinate) (PSAGE) and 60% of sebacic acid (SBA) copolymer (PSAGE-SBA60) and PSAGE and 80% of SBA copolymer (PSAGE-SBA80).

Sample	Molecular weight			Thermal properties					
	*M*_*n*_ (Da)	*M*_*w*_ (Da)	DP	*T*_*g*__1 (°C)	*T*_*g*__2 (°C)	*T*_*m*__1 (°C)	*T*_*m*__2 (°C)	*ΔH*_*m*__1 (J/g)	*ΔH*_*m*__2 (J/g)
PSAGE-SBA60	6400	8400	1.32	−38	−39	69	61	−96	−59
PSAGE-SBA80	9800	14,300	1.46	−38	−34	77	77	−110	−81

*M*_*n*_: number-average molecular weight; *M*_*w*_: weight-average molecular weight; DP: polydispersity; *T*_*g*_: glass transition (*T*_*g*__1, first heating cycle and *T*_*g*__2, second heating cycle); *T*_*m*_: melting point (*T*_*m*__1, first heating cycle and *T*_*m*__2, second heating cycle); *ΔH*_*m*_: melting enthalpy (*ΔH*_*m*__1, first heating cycle and *ΔH*_*m*__2, second heating cycle).

The thermal properties of PSAGE-SBA60 and PSAGE-SBA80 were evaluated using the DSC technique with two heating cycles (Fig. 2D and Table 3). The glass transition temperature (*T*_*g*_), typical of the amorphous phase, was the same for both polymers. In the second cycle, *T*_*g*__2 increased slightly for PSAGE-SBA80 and decreased slightly for PSAGE-SBA60. The melting temperature (*T*_*m*_), characteristic of crystalline materials, was the same in both cycles for PSAGE-SBA80. For PSAGE-SBA60, *T*_*m*_ was decreased from 69 to 61 °C. For both materials, the thermal effect absorbed much less energy in the second heating cycle, as the polymers did not have enough time to fully crystalize under the condition of enforced cooling between the cycles. This indicates the potential to adjust the crystallinity of polymers depending on the purpose, opening other possibilities for their application.

### 3.2 Physicochemical characterization of MPs

**Fig. 3:**
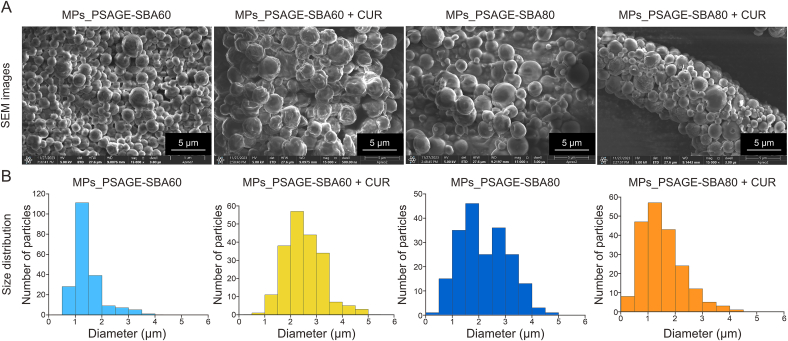
Morphology of microparticles (MPs) based on: poly(3-allyloxy-1,2-propylene succinate) (PSAGE) and 60% of sebacic acid (SBA) copolymer (MPs_PSAGE-SBA60), PSAGE and 80% of SBA (MPs_PSAGE-SBA80) copolymer, and MPs loaded with curcumin (CUR) copolymers (MPs_PSAGE-SBA60 + CUR and MPs_PSAGE-SBA80 + CUR, respectively): (A) scanning electron microscopy (SEM) images and (B) size distribution of MPs.

The empty and CUR-loaded MPs were successfully manufactured based on PSAGE-SBA60 and PSAGE-SBA80 copolymers using the oil-in-water emulsification/solvent evaporation method. All MPs obtained were spherical, as evidenced by SEM (Fig. 3A). The addition of CUR did not affect the shape of the MPs, but in the case of MPs_PSAGE-SBA60 + CUR, the MPs were slightly larger, while MPs_PSAGE-SBA80 + CUR were slightly smaller than empty MPs. The diameters of the MPs were measured on the basis of SEM images (Fig. 3B and Table 4). The size of empty MPs was in the range of 0.6–3.8 μm and 0.4–4.6 μm for MPs_PSAGE-SBA60 and MPs_PSAGE-SBA80, respectively. For MPs with CUR addition, the size ranged from 1.0 to 4.7 μm and from 0.2 to 4.2 μm for MPs_PSAGE-SBA60 + CUR and MPs_PSAGE-SBA80 + CUR, respectively. The span value (Table 4) showed that MPs_PSAGE-SBA80 and MPs_PSAGE-SBA80 + CUR were more polydisperse than MPs_PSAGE-SBA60 and MPs_PSAGE-SBA60 + CUR.

**Table 4:** Characterization of microparticles (MPs): median diameter, span, zeta potential, encapsulation efficiency (EE), and drug loading (DL).

Sample	Median diameter (μm)	Span	Zeta potential (mV)	EE (%)	DL (%)
MPs_PSAGE-SBA60	1.4	0.8	−24.3 ± 1.2	–	–
MPs_PSAGE-SBA80	2.0	1.2	−26.1 ± 0.5	–	–
MPs_PSAGE-SBA60 + CUR	2.4	0.7	−18.6 ± 2.2	21.2 ± 0.6	4.7 ± 0.1
MPs_PSAGE-SBA80 + CUR	1.3	1.5	−26.8 ± 2.4	17.7 ± 0.3	3.6 ± 0.1

–: no data. MPs_PSAGE-SBA60: MPs based on copolymer of poly(3-allyloxy-1,2-propylene succinate) (PSAGE) and 60% of sebacic acid (SBA) copolymer; MPs_PSAGE-SBA80: MPs based on copolymer of PSAGE and 80% of SBA copolymer; MPs_PSAGE-SBA60 + CUR: MPs_PSAGE-SBA60 loaded with curcumin (CUR); MPs_PSAGE-SBA80 + CUR: MPs_PSAGE-SBA80 loaded with CUR.

**Fig. 4:**
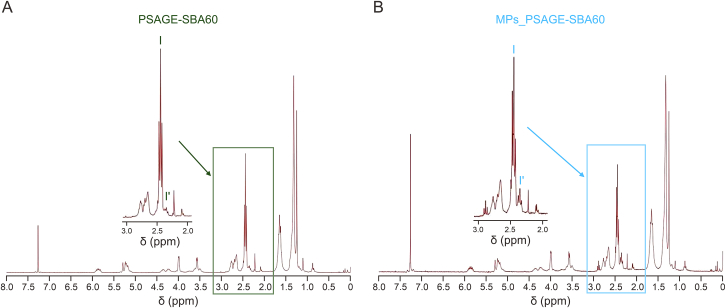
Proton magnetic nuclear resonance (^1^H NMR) spectra of (A) poly(3-allyloxy-1,2-propylene succinate) (PSAGE) and sebacic acid (SBA) copolymer (PSAGE-SBA60) and (B) microparticles (MPs) made of (MPs_PSAGE-SBA60) with the compared peaks marked. In the spectra of PSAGE-SBA80 and MPs_PSAGE-SBA80, the same peaks were found (data not shown). The insets indicate the spectra at higher magnification in the range 1.9–3.1 ppm. Signal assignment of protons: neighbouring the anhydride bond (I) and neighbouring the hydrolysed anhydride bonds to carboxylic acid (I').

Because polyanhydrides are easily degradable in an aqueous environment, even short contact with water causes their degradation. Therefore, we anticipated partial anhydride bond loss during the MPs manufacturing process. The ^1^H NMR technique allowed us to detect the signal originating from protons neighbouring the anhydride bond (marked in Fig. 4 as I). Anhydride bond hydrolysis to the carboxylic acid leads to a new peak shifted towards lower ppm values (marked in Fig. 4 as I′). Based on the ratio of integral values of both peaks before and after the fabrication of the MPs, the initial degradation as a result of polymer processing in the MPs was evaluated (Table 5). The results showed that PSAGE-SBA80 was approximately 7.5 times more resistant to the manufacturing process (only 1.1% loss of anhydride groups) than PSAGE-SBA60 (8.2% loss of anhydride groups).

**Table 5:** The ratio of condensed into anhydride carboxyl groups of the monomers before and after microparticles (MPs) fabrication.

Sample	Anhydride bonds (%)		Anhydride bonds loss (%)
	Initial polymer	MPs	
PSAGE-SBA60	89.3	82.0	8.2
PSAGE-SBA80	95.2	94.2	1.1

PSAGE-SBA60: copolymer of poly(3-allyloxy-1,2-propylene succinate) (PSAGE) and 60% of sebacic acid (SBA) copolymer; PSAGE-SBA80: copolymer of PSAGE and 80% of SBA copolymer.

The zeta potential measurements showed that all MPs were negatively charged (Table 4). The surface charge increased significantly for MPs_PSAGE-SBA60 + CUR compared to MPs_PSAGE-SBA60 and was equal to −18.6 ± 2.2 mV and −24.3 ± 1.2 mV, respectively. For MPs_PSAGE-SBA80 + CUR and MPs_PSAGE-SBA80, the zeta potential was similar and equal to −26.8 ± 2.4 mV and −26.1 ± 0.5 mV, respectively. The CUR addition to MPs_PSAGE-SBA80 did not influence the surface charge.

EE and DL were evaluated using the fluorometric method (Table 4). EE was equal to 21.1% ± 0.6% and 17.7% ± 0.3% for MPs_PSAGE-SBA60 + CUR and MPs_PSAGE-SBA80 + CUR, respectively. The DL for MPs_PSAGE-SBA60 + CUR and MPs_PSAGE-SBA80 + CUR was equal to 4.7% ± 0.1% and 3.6% ± 0.1%, respectively.

**Fig. 5:**
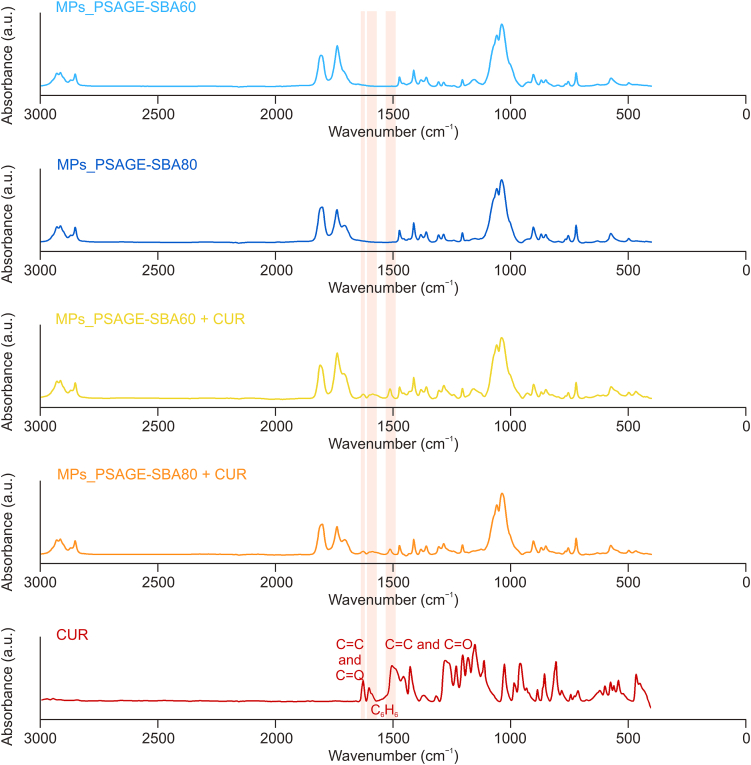
Fourier transform infrared (FTIR) spectra of unloaded microparticles (MPs) based on poly(3-allyloxy-1,2-propylene succinate) (PSAGE) and 60% of sebacic acid (SBA) copolymer (MPs_PSAGE-SBA60), MPs based on PSAGE and 80% SBA copolymer (MPs_PSAGE-SBA80), MPs loaded with curcumin (CUR) copolymers (MPs_PSAGE-SBA60 + CUR and MPs_PSAGE-SBA80 + CUR, respectively), and CUR.

Furthermore, the success of CUR encapsulation was confirmed by FTIR-ATR analyses (Fig. 5). Three additional bands were observed in the spectra of MPs_PSAGE-SBA60 + CUR and MPs_PSAGE-SBA80 + CUR compared to the unloaded MPs (MPs_PSAGE-SBA60 and MPs_PSAGE-SBA80). The most intense band with a maximum at 1506 cm^−1^ and the band with a maximum at 1625 cm^−1^ are due to C=C and C=O stretching vibrations of the CUR. The peak with a maximum at 1602 cm^−1^ comes from stretching vibrations of the benzene ring of the CUR [[31], [32], [33]].

### 3.3 Aerodynamic properties of MPs

The *d*_*aero*_ was calculated from the geometric diameter and the tapped density (Table 6). The lowest *d*_*aero*_ was obtained for MPs_PSAGE-SBA60 (0.66 μm), while the highest was obtained for MPs_PSAGE-SBA80 (1.14 μm). The flow character of the MPs was determined using the Carr index and Hausner ratio. The flowability of empty MPs was found to be passable for MPs_PSAGE-SBA60 and fair for MPs_PSAGE-SBA80. The addition of CUR improved the flowability of MPs_PSAGE-SBA80 + CUR compared to empty MPs, whereas it decreased the flowability of MPs_PSAGE-SBA60 + CUR.

**Table 6:** Flow character of microparticles (MPs).

Sample	Bulk density (g/cm^3^)	Tapped density (g/cm^3^)	*d*_*aero*_ (μm)	Carr index	Hausner ratio	Flow character
MPs_PSAGE-SBA60	0.18 ± 0.05	0.24 ± 0.05	0.66	24.8 ± 5.5	1.34 ± 0.10	Passable
MPs_PSAGE-SBA80	0.10 ± 0.02	0.13 ± 0.03	1.14	16.6 ± 4.0	1.20 ± 0.06	Fair
MPs_PSAGESBA60 + CUR	0.16 ± 0.02	0.22 ± 0.03	0.73	27.3 ± 2.0	1.38 ± 0.04	Poor
MPs_PSAGESBA80 + CUR	0.17 ± 0.03	0.20 ± 0.03	0.59	12.7 ± 2.1	1.15 ± 0.03	Good

*d*_*aero*_: theoretical aerodynamic diameter; MPs_PSAGE-SBA60: MPs based on copolymer of poly(3-allyloxy-1,2-propylene succinate) (PSAGE) and 60% of sebacic acid (SBA) copolymer; MPs_PSAGE-SBA80: MPs based on copolymer of PSAGE and 80% of SBA copolymer; MPs_PSAGE-SBA60 + CUR: MPs_PSAGE-SBA60 loaded with curcumin (CUR); MPs_PSAGE-SBA80 + CUR: MPs_PSAGE-SBA80 loaded with CUR.

**Fig. 6:**
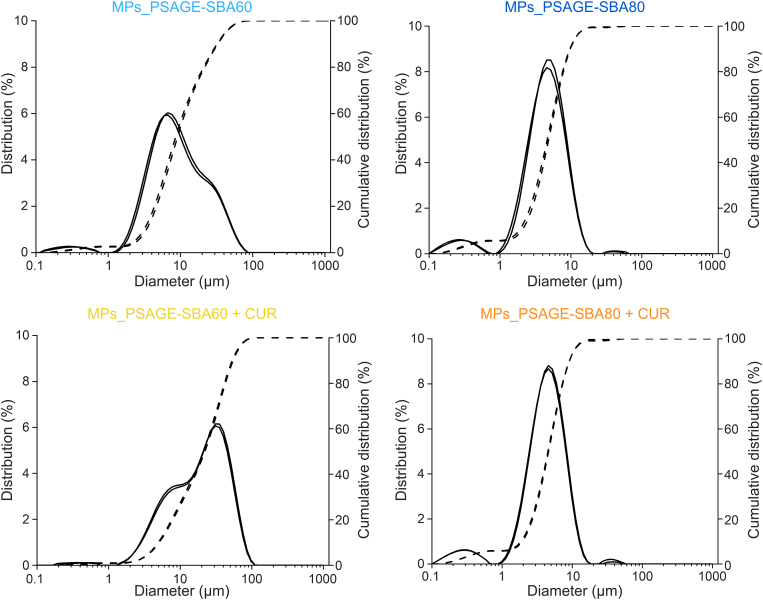
Size distributions of unloaded microparticles (MPs) based on poly(3-allyloxy-1,2-propylene succinate) (PSAGE) and 60% of sebacic acid (SBA) copolymer (MPs_PSAGE-SBA60), MPs based on based on PSAGE and 80% SBA copolymer (MPs_PSAGE-SBA80), and MPs loaded with curcumin (CUR) copolymers (MPs_PSAGE-SBA60 + CUR and MPs_PSAGE-SBA80 + CUR, respectively) obtained from the laser diffraction measurements.

Additionally, the size distribution of the MPs in the form of dry powder was measured using laser diffraction (Table 7 and Fig. 6). The median diameter (*d*_50_) was significantly different for both materials. For MPs_PSAGE-SBA80, it was equal to 4.75 ± 0.15 μm and for MPs_PSAGE-SBA60 it was 8.88 ± 0.37 μm. The addition of CUR did not affect the *d*_50_ of MPs_PSAGE-SBA80 + CUR compared to MPs_PSAGE-SBA80. The addition of CUR to MPs_PSAGE-SBA60 caused an increase of the *d*_50_ to 21.65 ± 0.64 μm. The *d* < 5 μm was similar for MPs_PSAGE-SBA80 and MPs_PSAGE-SBA80 + CUR and was equal to 53.39% ± 1.98% and 56.31% ± 0.91%, respectively. For MPs_PSAGE-SBA60 and MPs_PSAGE-SBA + CUR, *d* < 5 μm was significantly lower and was equal to 23.98% ± 0.10% and 9.99% ± 0.39%, respectively.

**Table 7:** Size of microparticles (MPs) measured by laser diffraction.

Sample	*d*_10_ (μm)	*d*_50_ (μm)	*d*_90_ (μm)	*d* < 5 μm (%)	Span
MPs_PSAGE-SBA60	3.26 ± 0.17	8.88 ± 0.37	32.45 ± 0.49	23.98 ± 1.91	3.29 ± 0.10
MPs_PSAGE-SBA80	1.93 ± 0.12	4.75 ± 0.15	9.76 ± 0.08	53.39 ± 1.98	1.65 ± 0.06
MPs_PSAGE-SBA60 + CUR	5.01 ± 0.11	21.65 ± 0.64	54.20 ± 1.13	9.99 ± 0.39	2.28 ± 0.02
MPs_PSAGE-SBA80 + CUR	1.90 ± 0.06	4.56 ± 0.07	9.09 ± 0.04	56.31 ± 0.91	1.58 ± 0.02

*d*_10_: fine fraction, the particle size threshold at which 10% of the sample is smaller; *d*_50_: median size, the particle size at which exactly half of the sample is smaller and half is larger; *d*_90_: coarsest fraction, the particle size threshold at which 90% of the sample is smaller; MPs_PSAGE-SBA60: MPs based on copolymer of poly(3-allyloxy-1,2-propylene succinate) (PSAGE) and 60% of sebacic acid (SBA) copolymer; MPs_PSAGE-SBA80: MPs based on copolymer of PSAGE and 80% of SBA copolymer; MPs_PSAGE-SBA60 + CUR: MPs_PSAGE-SBA60 loaded with curcumin (CUR); MPs_PSAGE-SBA80 + CUR: MPs_PSAGE-SBA80 MPs based on copolymer of PSAGE and 80% of SBA loaded with CUR.

### 3.4 CUR distribution within MPs

**Fig. 7:**
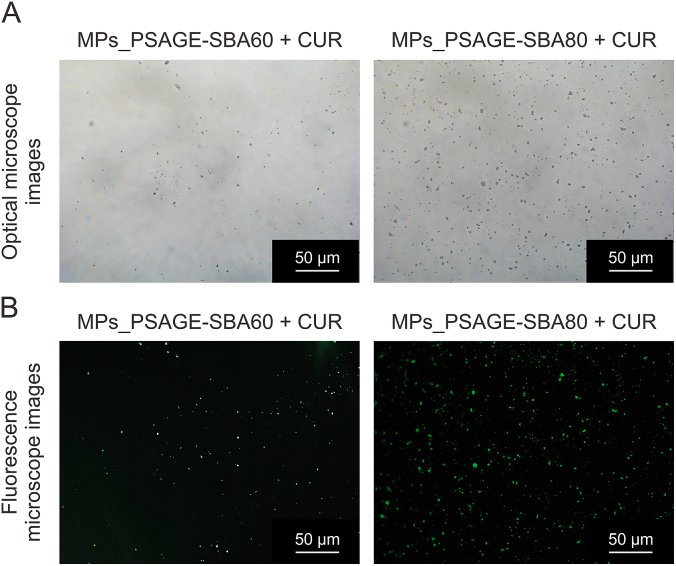
Microparticles (MPs) based on poly(3-allyloxy-1,2-propylene succinate) (PSAGE) and 60% of sebacic acid (SBA) loaded with curcumin (CUR) copolymer (MPs_PSAGE-SBA60 + CUR) and MPs based on PSAGE and 80% SBA loaded with CUR copolymers (MPs_PSAGE-SBA80 + CUR): (A) optical microscopy images and (B) CUR distribution in MPs observed under fluorescence microscopy.

Due to the fluorescence of CUR, its distribution in MPs was evaluated using fluorescence microscopy (Fig. 7). Images of MPs were first taken by optical microscopy (Fig. 7A) and then at the same place by fluorescence microscopy (Fig. 7B). All of the MPs visible under optical microscopy were also visible under fluorescence microscopy. In the case of larger MPs, the fluorescence signal was more intense; nevertheless, CUR was evenly distributed in both types of MPs.

### 3.5 Degradation study of MPs

**Fig. 8:**
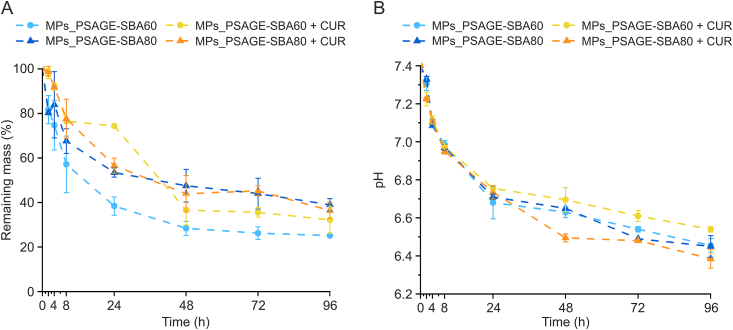
Degradation study in phosphate-buffered saline (PBS) up to 96 h of unloaded microparticles (MPs) based on poly(3-allyloxy-1,2-propylene succinate) (PSAGE) and 60% of sebacic acid (SBA) copolymer (MPs_PSAGE-SBA60), MPs based on PSAGE and 80% SBA copolymer (MPs_PSAGE-SBA80), and MPs loaded with curcumin (CUR) copolymers (MPs_PSAGE-SBA60 + CUR and MPs_PSAGE-SBA80 + CUR, respectively: (A) remaining mass of MPs and (B) pH of PBS after being in contact with MPs up to 96 h.

The degradation study of MPs (Fig. 8) was evaluated in PBS. Empty MPs_PSAGE-SBA60 lost its mass faster than MPs_PSAGE-SBA80. After 96 h, the mass was reduced to 25.2% ± 0.6% and 39.0% ± 2.8% for MPs_PSAGE-SBA60 and MPs_PSAGE-SBA80, respectively. However, the pH decreased similarly for both these samples. MPs_PSAGE-SBA60 + CUR and MPs_PSAGE-SBA60 + CUR were losing mass similarly for 8 h of incubation in PBS. After 8 h, MPs_PSAGE-SBA60 + CUR degraded slower, whereas after 24 h, it degraded faster than MPs_PSAGE-SBA80 + CUR. The value of pH was similar for 24 h for both types of samples. After that, the pH decreased faster for MPs_PSAGE-SBA80 + CUR than for MP_PSAGE-SBA60 + CUR.

### 3.6 In vitro cytotoxicity tests of MPs

**Fig. 9:**
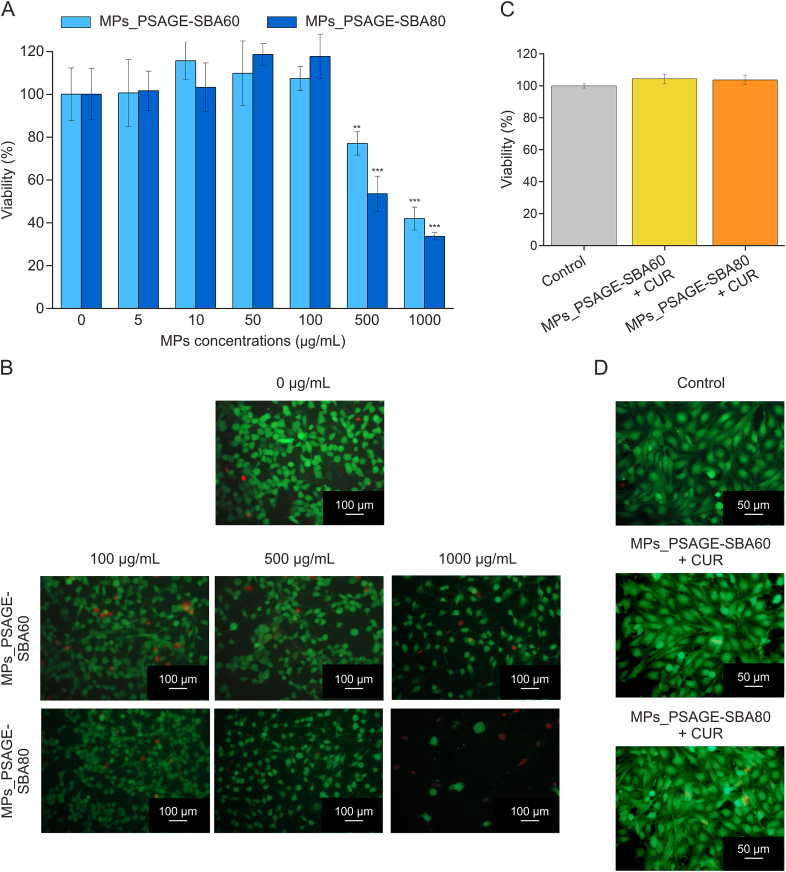
*In vitro* testing on microparticles (MPs). (A) Viability (evaluated by resazurin reduction assay) of human lung epithelial cells (BEAS-2B) treated with MPs based on poly(3-allyloxy-1,2-propylene succinate) (PSAGE) and 60% of sebacic acid (SBA) copolymer (MPs_PSAGE-SBA60) and MPs based on PSAGE and 80% of SBA copolymer (MPs_PSAGE-SBA80) at 5–1000 μg/mL concentrations. (B) Live/dead staining of BEAS-2B cells treated with MPs_PSAGE-SBA60 and MPs_PSAGE-SBA80 at 100, 500, and 1000 μg/mL. (C) Viability of BEAS-2B cells treated with curcumin (CUR)-loaded MPs: MPs_PSAGE-SBA60 + CUR and MPs_PSAGE-SBA80 + CUR at 100 μg/mL. (D) Live/dead staining of BEAS-2B cells treated with CUR-loaded MPs: MPs_PSAGE-SBA60 + CUR and MPs_PSAGE-SBA80 + CUR at 100 μg/mL. Control: cell culture in medium without MPs. ^∗∗^*P<* 0.01 and ^∗∗∗^*P<* 0.001.

The cytotoxicity of empty MPs was investigated in contact with BEAS-2B human lung epithelial cells. Cells were treated with MPs at concentrations ranging from 5 to 1000 μg/mL. Cell viability did not change significantly compared to the control (0 μg/mL) in the presence of 5–100 μg/mL for both types of MPs. At 500 μg/mL, the statistical difference compared to the control sample was observed at *P* *<* 0.01 and *P* *<* 0.001 for MPs_PSAGE-SBA60 and MPs_PSAGE-SBA80, respectively. Viability decreased to 77.1% ± 5.5% and 53.5% ± 8.3% for MPs_PSAGE-SBA60 and MPs_PSAGE-SBA80, respectively (Fig. 9A). The results were compatible with the live/dead staining images (Fig. 9B). Cells cultured with MPs at a concentration ≤ 100 μg/mL exhibited a typical morphology, the same as control (0 μg/mL MPs). Individual dead cells stained red were also visible. At higher concentrations, the number of cells was reduced.

Evaluation of the cytotoxicity of empty MPs allowed us to select a safe MPs dose equal to 100 μg/mL. The addition of MPs loaded with CUR at a concentration of 100 μg/mL had no effect on cell viability compared to untreated cells (Fig. 9C). There were no statistical differences between the samples. This observation was also confirmed by live/dead staining (Fig. 9D), in which the majority of the cells were stained green, thus alive.

### 3.7 Ex vivo cytotoxicity tests of MPs

**Fig. 10:**
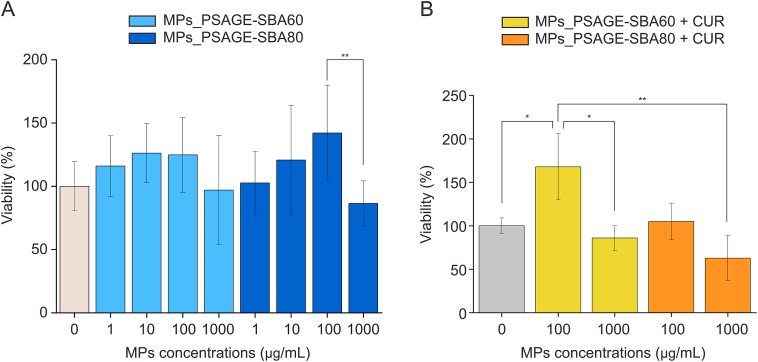
*Ex vivo* testing on microparticles (MPs). Viability of rat lung precision-cut tissue slices (PCTSs) after 24 h of incubation with (A) MPs based on poly(3-allyloxy-1,2-propylene succinate) (PSAGE) and 60% of sebacic acid (SBA) copolymer (MPs_PSAGE-SBA60) and MPs based on PSAGE and 80% of SBA copolymer (MPs_PSAGE-SBA80) suspensions at different concentrations from 1 to 1000 μg/mL and (B) in the suspensions of MPs based on PSAGE and 60% of SBA loaded with curcumin (CUR) copolymer (MPs_PSAGE-SBA60 + CUR) and MPs based on PSAGE and 80% of SBA loaded with CUR copolymer (MPs_PSAGE-SBA80 + CUR) suspensions at 100 and 1000 μg/mL. ^∗^*P<* 0.05 and ^∗∗^*P<* 0.01.

The viability of PCTS from rat lungs was normalized to the mean value of ATP/protein levels of the control samples. For each sample, 3 slices were used from the 3 different rats (9 PCTS in total, Fig. 10A). The negative impact of the MPs on the viability of the cells within the slices would be expressed by the lower ATP levels. However, no significant differences were observed between the control sample (incubated in pure medium without contact with MPs) and the samples incubated in the MPs suspensions at even 1000 μg/mL. A tendency of decreased viability was observed for the highest MPs concentrations; however, the differences were not statistically significant. SDs are relatively high, as the data were collected from different animals. Nevertheless, no drastic decrease in cell viability was observed. The only significant difference was found between MPs_PSAGE-SBA80 at 1000 and 100 μg/mL, which happened to have a slightly higher ATP-to-protein ratio. These observations confirmed the nontoxic concertation of MPs of 100 μg/mL. No drastic impact of MPs was observed even at the highest concentration of 1000 μg/mL.

To assess the potential influence of CUR on PCTS viability, the experiment was carried out on MPs_PSAGE-SBA60 + CUR and MPs_PSAGE-SBA60 + CUR at 100 and 1000 μg/mL (Fig. 10B). In this case, no sample showed significantly lower ATP values than the control. However, both materials showed significant differences between concentrations of 100 and 1000 μg/mL of MPs_PSAGE-SBA60 + CUR. A similar difference was noticed between MPs_PSAGE-SBA60 + CUR at 100 μg/mL and the control. Apart from the latter sample, no impact of CUR on PCTS viability was observed. Again, the SDs are relatively high, so it may not be claimed that there was no toxicity, but definitely no drastic toxic effect was caused by the MPs.

## 4 Discussion

Polyanhydrides are a group of rapidly degradable polymers that show promising properties for pulmonary drug delivery and are expected to release APIs before removal from the respiratory tract by clearance mechanisms. Herein, we synthesized and characterized copolymers of PSAGE and SBA, and we tested whether MPs of PSAGE-SBA may be carriers of CUR. The physicochemical properties and cytotoxicity of the MPs were also evaluated.

The copolymers of PSAGE and SBA were successfully obtained by melt-polycondensation, which was confirmed by ^1^H NMR and FTIR analyses. The ^1^H NMR analysis of the copolymers allowed us to identify the peaks that were consistent with the results presented in the literature for pure PSAGE [27], and also for its copolymers with aliphatic dicarboxylic acids [28]. In addition, the FTIR spectra showed characteristic bands for PSAGE and SBA. Generally, polyanhydrides belong to the class of hydrophobic polymers [34]. In our work, we observed that the addition of 40% PSAGE made the resulting copolymer more hydrophilic. The hydrophilicity of the materials has some advantages in pulmonary drug delivery, since MPs made from a more hydrophilic polymer are known to better avoid uptake by macrophages [35] and better penetrate the mucus layer [36]. Degradation of polyanhydrides depends, among others, on hydrophilicity [37]. The degradation study of copolymers carried out by Jaszcz and Łukaszczyk [28] evidenced that PSAGE-SBA60 lost its mass faster than PSAGE-SBA80. After five days, the mass of copolymers was reduced by approximately 30% and 50% for PSAGE-SBA80 and PSAGE-SBA60, respectively. The same tendency was observed in our study during the degradation of MPs. Additionally, the SFE of polymers also has an effect on the properties of MPs. The encapsulation of APIs in a polymeric matrix with low SFE results in a better dispersion of API within the MPs [38].

The MPs were successfully obtained using emulsification with solvent evaporation method. During the MPs manufacturing process, the anhydride bonds are partially hydrolysed. In our study, we calculated that during the production of MPs_PSAGE-SBA60, about 8% of the anhydride bonds are hydrolysed, in contrast to MPs_PSAGE-SBA80, where only 1% of the anhydride bonds are lost. A similar method was used by Jaszcz [21] who determined the loss of anhydride bonds during the degradation of PSAGE-SBA60 and PSAGE-SBA80. The results showed that PSAGE-SBA60 hydrolysed faster than PSAGE-SBA80, which is consistent with our observations.

The success of MPs manufacturing was confirmed by SEM. The manufactured MPs were spherical, similar to our previous work [22], where the MPs were made of nonmodified PSBA. The MPs had a smooth surface, and only MPs_PSAGE-SBA60 + CUR had a slightly wrinkled surface. The geometric diameter was also determined from the SEM images. However, it is very often different from *d*_*aero*_, which determines the deposition of MPs in the respiratory tract [4]. The ideal *d*_*aero*_ for inhalation is between 1 and 5 μm [3,4]. All obtained MPs had a geometric diameter smaller than 5 μm, whereas the laser diffraction measurements showed that the MPs diameters were significantly higher. Depending on the material used, the fraction of MPs smaller than 5 μm was approximately 10% and 57% for MPs_PSAGE-SBA60 + CUR and MPs_PSAGE-SBA80 + CUR, respectively. The observation is related to the permanent agglomeration of the MPs during the manufacturing, which may not be seen in the SEM pictures. These results are compatible with the flow character determined on the basis of the Carr index and the Hausner ratio. We assume that such a large difference in permanent agglomeration is a result of the initial degradation, which occurs mainly on the surface of the MPs. The degraded surface may cause an easier fusion of the MPs. The similar *d*_50_ measured by laser diffraction for polysaccharide MPs loaded with sodium cromoglycate was determined by Gallo et al. [39]. However, the flowability of their MPs was classified as poor, which is related to the higher value of bulk and tapped density compared to our MPs. Ceschan et al. [40] manufactured MPs from polylysine and dextran with similar geometric diameters. In that case, they obtained a fraction *d* < 5 μm in the range of 39%–50%, depending on the formulations. They also calculated the Carr index, however, their powder had very poor flowability. Although they observed that the mixing of MPs and lactose in a 1:3 ratio significantly improved the flow character of the formulation.

The zeta potential of the MPs was also particularly important. Negatively charged MPs are repelled and thus show a lower tendency for agglomeration, whereas positively charged MPs bind to the mucus in the airways. MPs with a neutral charge are considered beneficial for mucus penetration [41]. Furthermore, charged MPs are more readily to be uptaken by macrophages [35]. Our MPs are negatively charged. CUR addition to MPs_PSAGE-SBA60 increases the zeta potential towards the neutral values. Therefore, MPs_PSAGE-SBA60 + CUR should be able to better penetrate the mucus layer and reach endothelial cells or bacteria located under the mucus layer [42].

EE and DL were determined using the fluorometric method and FTIR analyses, where three additional peaks were observed for CUR-loaded MPs. The highest EE and DL were determined for MPs_PSAGE-SBA60 + CUR. In our study, we confirmed that CUR is uniformly distributed within MPs. In the literature, there are only a few articles on CUR encapsulation in polyanhydrides. Lv et al. [43] encapsulated CUR in 4-arm PEG-block-poly(anhydride-esters) micelles with DL equal to 7.0% ± 0.2%. In their next study [44], the DL increased to 7.62% ± 0.11% by changing the 4-arm PEG for PEG with a molecular weight of 2000 Da. In our study, the DL depended on the PSAGE to PSBA feed ratio. It suggests that increasing the PSAGE ratio in the copolymer can improve the DL.

The potential use of MPs as drug carriers requires the determination of their cytotoxicity. Cytotoxicity was evaluated in contact with BEAS-2B lung epithelial cells of non-malignant origin. The empty MPs obtained from both copolymers were non-cytotoxic at a concentration of 100 μg/mL. In our previous work [22], the MPs manufactured from PSBA were cytotoxic at a concentration higher than 50 μg/mL in contact with BEAS-2B lung epithelial cells. Both experiments were performed under the same conditions, which proves that MPs manufactured from PSAGE-SBA copolymers are less cytotoxic than the MPs made from pure PSBA. Cytotoxicity tests performed *ex vivo* on PCTS confirmed the safe concentration of 100 μg/mL. Interestingly, the decrease in viability of the cells within PCTS was much lower than during *in vitro* tests, although the *ex vivo* model is believed to be more sensitive to toxic compounds [45]. The key parameter that influenced viability seems to be associated with the incubator movement. In static *in vitro* conditions, MPs sediment and cover cells grown in cell culture wells, while in the *ex vivo* model, constant shaking keeps them floating throughout the incubation. PCTS float in the medium as well and remain in contact with the MPs; however, the exposure of the cells to the MPs is dynamic. Also, *in vitro* cell culture concerns only one type of cells, whereas the PCTS models include all the cells that are present in the lung tissue.

We encapsulated CUR in PSAGE-SBA copolymers for the first time. CUR has previously been encapsulated in polyanhydrides by Lv et al. [43,44], but those MPs have not been designed as pulmonary drug carriers. In our study, we developed PSAGE-SBA MPs dedicated to pulmonary delivery for the first time. Our MPs were spherical with optimal size and aerodynamic properties for lung deposition. Significant differences were observed between two polymers tested. The results obtained showed that PSAGE-SBA80 was more suitable for the fabrication of inhalable drug delivery carriers for CUR. In particular, it was characterized by almost negligible degradation and anhydride loss during MPs fabrication and significantly better aerodynamic properties.

Although the flow character and laser diffraction results are promising, the MPs behaviour in lungs could be different, because the MPs powder may behave differently in a dry powder inhaler. That is why more advanced aerodynamic studies are required. Therefore, additional test of aerosolization and a more detailed study with a next generation impactor (NGI) will be beneficial in determining the potential of the MPs to properly deposited in the lungs. The MPs degrade fast by surface erosion and release encapsulated CUR. According to Domb and Nudelman [46], the solubility of polyanhydride degradation products determines the drug release rate. However, this assumption will need to be confirmed. It is unlikely for the MPs to lose their mass without releasing the encapsulated substance, but it does not inform about the kinetics of the release process, which will need to be evaluated before implementation. *In vitro* and *ex vivo* tests showed that PSAGE-SBA MPs are less cytotoxic than MPs manufactured from pure PSBA [22]. Also, the antibacterial properties of our MPs should be confirmed in the future. Additionally, more advanced *in vitro* tests such as reactive oxygen species (ROS) and cytokines production are required. These tests will allow us to characterize the immune response to our MPs to ensure their safety and efficacy before *in vivo* testing and clinical trials.

## 5 Conclusions

The MPs loaded with CUR manufactured from PSAGE-SBA copolymers have the potential as dry powder formulations to improve the treatment of lung diseases. The size, morphology, and degradation rate were found to be suitable for this application. The laser diffraction measurements and flowability showed promising results for MPs_PSAGE-SBA80 + CUR. The MPs were also not cytotoxic, as shown in contact with lung epithelial cells *in vitro* and in rat lung tissue *ex vivo* model. However, prior to preclinical tests, MPs must be investigated in terms of antibacterial and anti-inflammatory properties, mucus penetration, and uptake by macrophages.

## CRediT authorship contribution statement

**Karolina Knap:** Writing – original draft, Software, Methodology, Investigation, Conceptualization. **Konrad Kwiecień:** Writing – review & editing, Methodology, Investigation, Conceptualization. **Jonasz Czajkowski:** Methodology, Investigation. **Rafał Szostecki:** Methodology, Investigation. **Daria Niewolik:** Methodology, Investigation. **Katarzyna Jaszcz:** Validation, Resources. **Peter Olinga:** Validation, Methodology. **Katarzyna Reczyńska-Kolman:** Writing – review & editing, Validation, Supervision, Conceptualization. **Elżbieta Pamuła:** Writing – review & editing, Validation, Supervision, Resources, Project administration, Funding acquisition, Formal analysis, Data curation, Conceptualization.

## Declaration of competing interest

The authors declare that they have no known competing financial interests or personal relationships that could have appeared to influence the work reported in this paper.
